# Cerebral Microdialysis Monitoring to Improve Individualized Neurointensive Care Therapy: An Update of Recent Clinical Data

**DOI:** 10.3389/fneur.2017.00601

**Published:** 2017-11-13

**Authors:** Laurent Carteron, Pierre Bouzat, Mauro Oddo

**Affiliations:** ^1^Department of Anesthesiology and Intensive Care Medicine, University Hospital of Besançon, University of Bourgogne – Franche-Comté, Besançon, France; ^2^Department of Anesthesiology and Critical Care, University Hospital Grenoble, Grenoble, France; ^3^Department of Intensive Care Medicine, Centre Hospitalier Universitaire Vaudois (CHUV), University of Lausanne, Lausanne, Switzerland

**Keywords:** microdialysis, traumatic brain injury, subarachnoid hemorrhage, cerebral metabolism, ischemia, hypoxia, biomarkers, neurointensive care

## Abstract

Cerebral microdialysis (CMD) allows bedside semicontinuous monitoring of patient brain extracellular fluid. Clinical indications of CMD monitoring are focused on the management of secondary cerebral and systemic insults in acute brain injury (ABI) patients [mainly, traumatic brain injury (TBI), subarachnoid hemorrhage, and intracerebral hemorrhage (ICH)], specifically to tailor several routine interventions—such as optimization of cerebral perfusion pressure, blood transfusion, glycemic control and oxygen therapy—in the individual patient. Using CMD as clinical research tool has greatly contributed to identify and better understand important post-injury mechanisms—such as energy dysfunction, posttraumatic glycolysis, post-aneurysmal early brain injury, cortical spreading depressions, and subclinical seizures. Main CMD metabolites (namely, lactate/pyruvate ratio, and glucose) can be used to monitor the brain response to specific interventions, to assess the extent of injury, and to inform about prognosis. Recent consensus statements have provided guidelines and recommendations for CMD monitoring in neurocritical care. Here, we summarize recent clinical investigation conducted in ABI patients, specifically focusing on the role of CMD to guide individualized intensive care therapy and to improve our understanding of the complex disease mechanisms occurring in the immediate phase following ABI. Promising brain biomarkers will also be described.

## Introduction

Cerebral microdialysis (CMD) has progressively evolved from a tool for clinical research into an additional brain monitoring modality to guide neurointensive care (1, 2). Evidence has accrued over the last years that CMD monitoring—in combination with other modalities such as intracranial pressure (ICP) and brain tissue PO_2_ (PbtO_2_), so called multimodal monitoring—may help guiding individualized intensive care therapy of comatose brain-injured patients, mainly after traumatic brain injury (TBI) and aneurysmal subarachnoid hemorrhage (SAH) (3, 4). Clinical utility of CMD has been particularly shown for the management of “secondary” cerebral insults, i.e., the number of pathological events that occur in the early phase following acute brain injury (ABI). The use of CMD has contributed to better define therapeutic thresholds for several routine interventions, such as cerebral perfusion pressure (CPP) optimization, oxygen therapy, red blood cell transfusion (RBCT), and metabolic control (blood glucose and nutrition). Exploration of the injured brain with CMD has also greatly contributed to better understand important post-injury mechanisms—such as energy dysfunction, hyperglycolysis, cortical spreading depressions, subclinical seizures, or brain edema—and to identify potential novel biomarkers of injury and prognosis. Recent reviews focused on specific technical aspects related to CMD monitoring, both in terms of the catheters and microdialyzate analyser technology (1). The scope of this review was to summarize recent clinical investigation conducted in neurocritical care patients, aiming to discuss the role of CMD to guide individualized intensive care therapy and to improve our understanding of the complex disease mechanisms occurring in the immediate phase following severe brain injury. We also describe emerging data on the potential utility of CMD to assess novel biomarkers of injury, as well as its role in interventional and pharmacological studies. We mainly focused our review on clinical studies published during the last 5 years (January 2012 to September 2017) and performed in patients with ABI, including TBI, SAH, and ICH.

## Interpretation of CMD Variables and Reference Values

In clinical practice, CMD biomarkers (generally sampled every hour and immediately analyzed at the bedside) should always be interpreted in the context of monitor location, type of injury, and patient clinical condition. Based on accrued clinical data over the last decade linking glucose and lactate/pyruvate (L/P) ratio with principal outcomes after ABI, compared to glutamate and glycerol, the 2015 CMD Consensus proposed to interpret CMD biomarkers in a tiered fashion and to use primarily CMD L/P ratio and glucose as step 1 to guide clinical interventions (2). Abnormalities of CMD L/P ratio and glucose reflect the complex pathophysiology underneath ABI; therefore, correct interpretation require integration of other monitored variables such as ICP and PbtO_2_.

Elevated CMD lactate and L/P ratio may be a marker of inadequate cerebral blood flow (CBF) and/or oxygen delivery. In this context, dramatic increases may be observed, which are associated with a concomitant decrease in CMD pyruvate and glucose. Given that cerebral circulation and/or oxygenation are impaired, ICP/CPP and/or PbtO_2_ values will be abnormal.

However, CMD lactate and L/P ratio may be elevated because of other mechanisms than ischemia or hypoxia (5). Cerebral energy dysfunction/failure has been described despite CBF and brain tissue oxygenation being normal (6, 7), whereby elevations of CMD lactate and L/P ratio may be predominantly attributable to increased glycolysis or mitochondrial dysfunction (impairment of oxygen utilization or cytopathic hypoxia) (8, 9). In this context, pyruvate may be normal or elevated, and elevations of CMD lactate and L/P ratio are of a lesser extent than during frank ischemia/hypoxia.

Low CMD glucose, therefore, may be related to cerebral energy dysfunction (10). On the other hand, apart from cerebral causes (ischemia/hypoxia or energy dysfunction), inadequate systemic glucose, because of intensive insulin therapy to maintain strict glycemic control, may cause further reductions of CMD glucose (11, 12).

To direct individualized intensive care therapy, it is therefore important to consider CMD L/P ratio rather than lactate alone, to look for dynamic changes and trends of both CMD L/P ratio and glucose, and finally to take into account additional monitor modalities (ICP/PbtO_2_), according to the modern paradigm of multimodality monitoring (13, 14).

Interpretation of absolute values is also dependent on probe location in an area of normal-appearing vs. around a lesion (e.g., hematoma or contusion) (2, 15). Also, a recent study in SAH patients suggests that delayed cerebral ischemia may be detected only when the probe is located within a brain area later affected by secondary infarction, which may justify the use of implantation guidelines (16).

**Figure 1:**
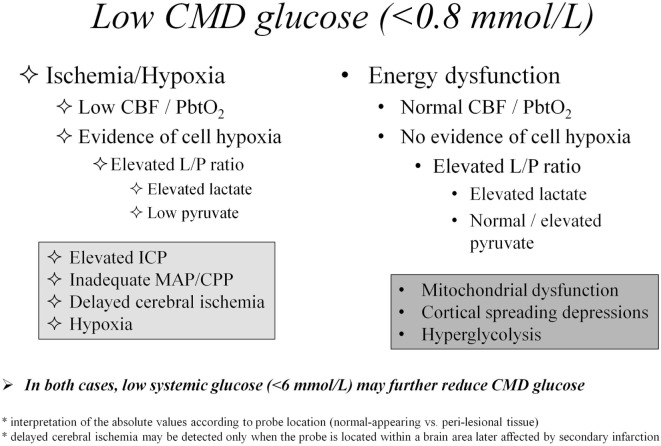
Differential diagnosis of cerebral metabolic abnormalities based on cerebral microdialysis. Abbreviations: CBF, cerebral blood flow; CMD, cerebral microdialysis; CPP, cerebral perfusion pressure; ICP, intracranial pressure; L/P, lactate/pyruvate; MAP, mean arterial pressure; PbtO_2_, brain tissue oxygen pressure.

In Figure 1, we propose an algorithm for interpretation of CMD abnormalities, centered on low CMD glucose as starting point of the clinical reasoning.

As for reference values, L/P ratio >25 is considered abnormal (impaired cerebral oxidative metabolism), while L/P ratio >40 is the critical level above which brain energy crisis is defined. The reference level for CMD glucose is still debated, but probably lies at 1 (±0.15) mmol/L (17).

## CMD to Guide Individualized Intensive Care Therapy

### Optimization of Substrate Supply

The CMD technique allows semicontinuous monitoring of cerebral glucose metabolism and of the interactions between blood and brain glucose in humans under conditions of varying glycemia (18). Glucose is the main substrate for the brain. However, in the aftermath of injury, the brain’s ability to use glucose may be reduced (19). Cerebral extracellular glucose may be limited (10, 20), therefore, enabling adequate glucose supply in ABI patients appears crucial to attenuate further brain damage (21). Following the two large single-center studies by van Den Berghe and colleagues in the early 2000 (22, 23), suggesting that tight glycemic control may benefit general critically ill patients, Vespa and colleagues were the first to show that actually this so-called intensive insulin strategy was associated with an increased prevalence of low CMD glucose and elevated LPR (24). This CMD study was concomitant to another outcome study by the Leuven’s group showing that, at the contrary, strict glycemic control may also benefit the outcome of neurointensive care patients (25). Additional CMD studies from several groups subsequently confirmed the seminal clinical investigation by Vespa and colleagues, showing that indeed strict glycemic control might reduce cerebral glucose availability and aggravate cerebral energy dysfunction (11, 26–31). Given the results of the multicentre NICE-SUGAR study, which did not confirm substantial outcome benefit for intensive vs. moderate blood glucose control both in the general ICU population (32, 33), and in the *post hoc* analysis of neurotrauma patients (34), a strategy of liberal glycemic control (7–10 mmol/L) was generally felt as safer in critically neurological patients by international recommendations (35). Indeed, using a cross-over design that alternated tight to moderate glycemic control, Vespa confirmed previous findings that intensive insulin therapy was associated with increased metabolic distress, as judged by lower CMD glucose and higher CMD L/P ratio during tight glycemia (12).

The glycemic control controversy illustrates how CMD monitoring has contributed to the actual progresses of intensive care therapies, and how physiologically oriented studies may influence our practice, especially in the field of neurointensive care where “true” evidence-based medicine derived from RCT is often lacking. A recent example of such approach was provided by the Innsbruck group led by Helbok: the authors found that rapid effective institution of enteral nutrition was associated with an increase in CMD glucose that was directly dependent on the magnitude of increase of blood glucose (36), reinforcing the recommendations for the early institution of enteral feeding in neurointensive care patients.

The Consensus on CMD suggests the use of CMD monitoring for the detection and treatment of low cerebral glucose, and to guide systemic glucose management and insulin use (2).

### Optimization of Cerebral Perfusion

CMD markers—such as glucose and L/P ratio—may be good surrogate markers of CBF, and indeed this has recently been confirmed by several clinical studies combining microdiaylsis with brain imaging, both in patients with SAH (37–39) and TBI (40). A recently published small observational cohort study illustrated the potential value of CMD monitoring to help detecting cerebral hypoperfusion in comatose aSAH patients, in whom, the clinical examination was unreliable (37). This study stressed the importance of following dynamic trends over time of both CMD L/P ratio and glucose for the timely detection of secondary cerebral ischemic insults. It also confirmed the potential value of CMD biomarkers to avoid low CPP by adjusting CPP thresholds individually in comatose ABI patients (16, 41–43). Indeed, Bouzat and colleagues found that the addition of CMD (in combination with PbtO_2_) to ICP monitoring significantly improved the accuracy of detecting secondary hypoperfusion in patients with severe TBI (40).

The use of CMD monitoring to optimize CCP in order to prevent/avoid ischemia is recognized as potentially clinically useful for TBI and SAH patients by the Consensus on CMD (2).

### Optimization of Oxygen Transport: Blood Transfusion and Oxygen Therapy

#### Red Blood Cell Transfusion

Whether restrictive or more liberal thresholds for hemoglobin and RBCT should be used in neurointensive care is still debated, given the lack of randomized clinical trials in this setting. It is possible that the therapeutic approach may vary individually, according to the extent of injury; therefore, patients with more severe brain insults may benefit from higher hemoglobin (Hgb) levels (44, 45). Indeed, low Hgb <9 g/dL was shown to be associated with increased CMD markers of cerebral ischemia (elevated L/P ratio and low CMD glucose) (46, 47). The question is whether enhancing cerebral oxygen transport with RBCT may reduce cerebral damage: RBCT might improve PbtO_2_ in the majority (although not all) of patients (48, 49); however, improved PbtO_2_ did not translate into a clinically relevant benefit on cellular metabolism, as quantified by the non-significant amelioration of CMD L/P ratio (50, 51).

#### Oxygen Therapy

In various subsets of critically ill patients, including those with ABI, increasing inspired fraction of oxygen (FiO_2_) to achieve arterial hyperoxia (arterial partial pressure of oxygen, PaO_2_, >150 mmHg) was associated with worse outcome (52). Whether or not hyperoxia is beneficial after ABI remains controversial. Physiological studies testing the effect of hyperoxia on CMD biomarkers were conducted predominantly on TBI patients. Improving PbtO_2_ by way of normobaric hyperoxia may reduce L/P ratio (53, 54), although this effect seems of limited clinical relevance (55). When using CMD glutamate as a marker of increased excitotoxicity, Quintard and colleagues found an association between normobaric hyperoxia and increased cerebral glutamate (56). Recently, two prospective single-center trials brought additional important insights. Ghosh and colleagues, testing 120-min normobaric hyperoxia challenge in the acute phase (24–72 h) of TBI (16 patients; using an advanced multimodal monitoring, including PbtO_2_, CMD, near-infrared spectroscopy, and transcranial Doppler) found that hyperoxia was associated with an improvement of L/P ratio, as well as all other oxygenation and perfusion parameters, consistent with increased aerobic cerebral metabolism and better cellular redox state (57). Vidal-Jorge and colleagues in an elegant study using CMD to sample biomarkers of oxidative stress (8-iso-Prostaglandin F2α) found that increasing FiO_2_ to 1.0 for 4 h resulted in marked reduction in both CMD lactate and CMD L/P ratio only in patients with more severe injury, as defined by a CMD lactate >3.5 mmol/L, but did not change energy metabolism in the whole group of patients (58). Furthermore, hyperoxia caused a significant increase in 8-iso-PGF2α in patients in whom oxidative stress was detected at baseline, but not in those without (58).

Rockswold and colleagues, using a Phase II observational design, found that hyperbaric oxygen therapy [1 h at 1.5 atmospheres absolute (ATA)], followed by 3-h normobaric hyperoxia (100% FiO_2_ at 1.0 ATA) was effective in improving CMD L/P ratio and glycerol after TBI, both in relatively uninjured brain as well as in peri-contusional tissue; tissue benefit translated into better outcome in this study (59).

Overall, CMD has evolved over time as a tool that may help guiding individualized targeted therapy at the bedside in ABI patients and to test the physiologic response to a specific intervention (Table 1).

**Table 1:** Examples of ICU interventions guided by CMD.

	Energy supply		Cerebral Perfusion	Oxygen transport	
				FiO_2_, PaO_2_	(Hgb)
Therapeutic intervention	Insulin therapy	Enteral nutrition	Intracranial pressure/CPP targets	NBHO	RBCT
Risks	↓ CMD glucose <0.7 mmol/L	↑ blood glucose	Ischemia, ↓ CPP	Increased excitotoxicity	Ischemia/hypoxia vs. RBCT-related complications
Benefits	Optimal glycemia	↑ CMD glucose	Optimal CPP	Optimal PaO_2_	Optimal (Hgb)
CMD targets	CMD glucose >0.7 mmol/L		↓ L/P ratio	↓ L/P ratio	↓ L/P ratio
			↑ CMD glucose		

*CMD, cerebral microdialysis; CPP, cerebral perfusion pressure; Hgb, hemoglobin; FiO_2_, fraction of inspired oxygen; ICU, intensive care unit; L/P lactate/pyruvate; NBHO, normobaric hyperoxia; PaO_2_, arterial partial pressure of oxygen; RBCT, red blood cell transfusion*.

### CMD to Test the Efficacy of Pharmacological Interventions

Although it was not validated so far in large multicentre studies, CMD biomarkers such as CMD L/P ratio and glucose are associated with patient prognosis, at least in TBI patients (60). Therefore, it is conceivable to use CMD metabolites as surrogate outcome endpoints to test therapeutic efficacy in Phase II clinical trials.

Examples of therapies tested in studies using CMD biomarkers as surrogate outcome endpoints include:

– nitric oxide synthase inhibition (61)
– recombinant human interleukin-1 receptor antagonist (62)
– antiepileptic drugs (63, 64)
– focally perfused succinate (65)
– intravenous hypertonic lactate (66, 67)
– sedation (68).

Measuring the concentrations of drug molecules in the brain extracellular fluid appears superior to cerebrospinal fluid or plasma to test the ability to effectively deliver pharmacological agents across the blood–brain barrier into the brain and is an important step in the development of central nervous system therapies. CMD sampling can give valuable pharmacokinetic information of variations with time in drug concentrations of brain interstitial tissue versus plasma and may help in designing future therapies (69, 70), or to test drug penetration of several pharmacologic agents, such antimicrobials (71, 72) or antiepileptic drugs (63, 64).

## CMD to Explore the Complex ABI Pathophysiology

**Figure 2:**
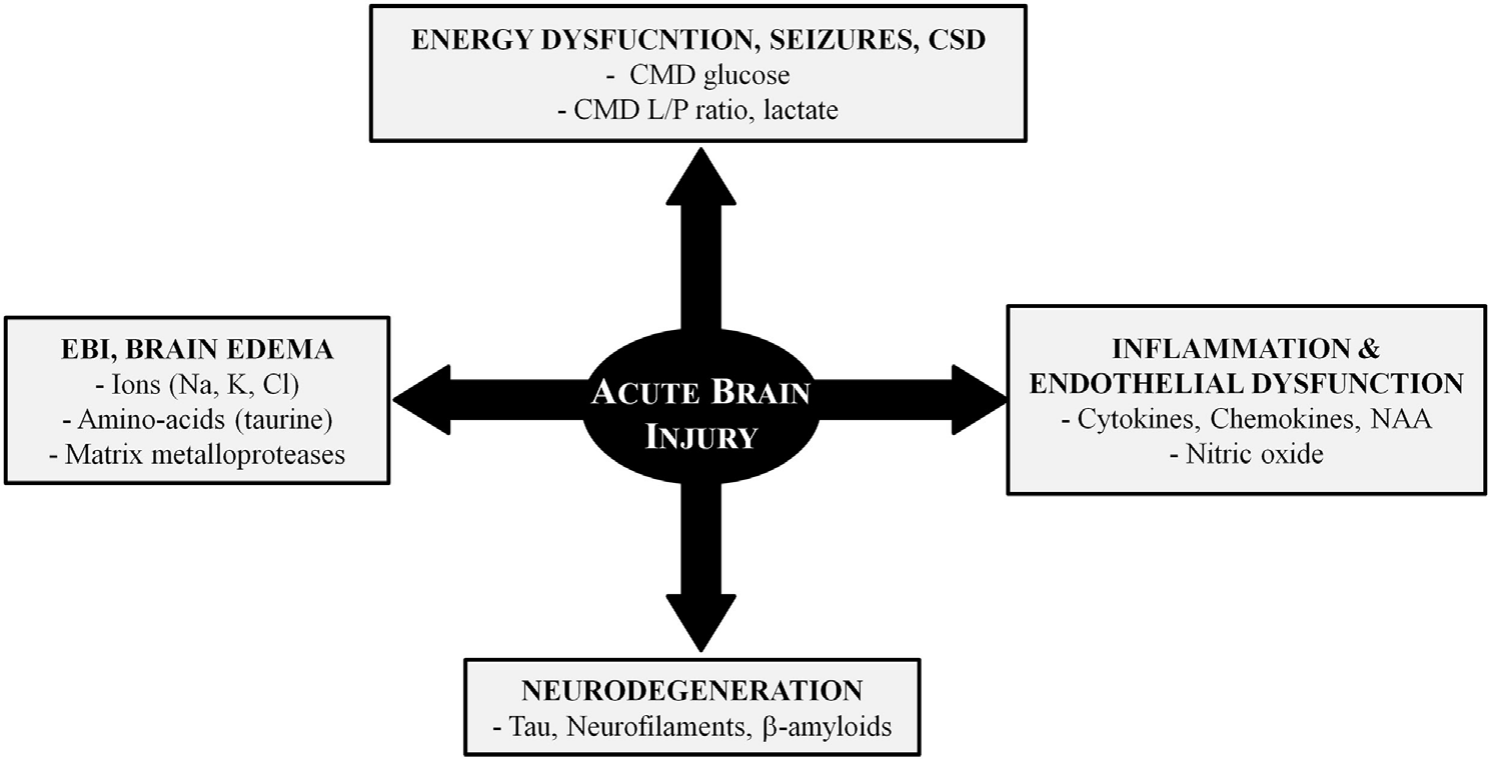
Pathophysiology of acute brain injury: the role of cerebral microdialysis. Abbreviations: CMD, cerebral microdialysis; CSD, cortical spreading depressions; EBI, early brain injury; L/P, lactate/pyruvate; NAA, n-acetyl aspartate.

Alterations of cerebral perfusion/oxygenation (73–75) and brain energy metabolism (9, 19, 20, 76–82) are important determinants of ABI. However, additional mechanisms are implicated in post-injury pathophysiology and CMD has contributed to elucidate some of these mechanisms (Figure 2). In this context, CMD catheters with larger membrane cut-off (100 kDa) than the standard ones (20 kDa) may have great utility for the identification and bedside follow-up of biomarkers of injury (e.g., cytokines, metallo-proteases) and recovery (e.g., markers of neurodegeneration) in specific pathologies (70, 83).

### The Link between Energy Dysfunction and Electrographic Crisis

Non-convulsive seizures and pseudo-periodic discharges might amplify secondary cerebral damage in the setting of ABI: using an elegant approach combining CMD with surface and intra-cortical electro-encephalography, Vespa and colleagues recently established a mechanistic link between seizures and metabolic crisis (84). This study is another example of how CMD can be used to monitor complex and concealed mechanisms but also to test the efficacy of future interventions aimed at specifically targeting seizure suppression.

Along the same line, pathological spreading depressions, which are frequently seen in TBI and SAH patients (85), cause significant local cerebral metabolic disturbances (reduced CMD glucose, elevated CMD LPR, and glutamate) (86–88); therefore, it is conceivable to use CMD as target for future interventional trials aimed at specifically treating spreading depressions.

### Early Brain Injury and Cerebral Edema

Microdialysis studies have contributed to better characterize the exact nature of cerebral edema in different pathologies and to differentiate between cellular (or cytotoxic) and vasogenic edema. Alterations in the ionic profile of the extracellular space [main electrolytes (Na^+^, K^+^, Cl^−^) and amino-acids like taurine] correlate with cellular edema in patients with diffuse injury after TBI (89–92). Matrix metalloproteases (MMP) are important pathogenic determinants of blood–brain barrier breakdown and vasogenic edema: using 100 kDa catheters, which allows sampling of larger molecules, elevated CMD MMP have been observed in patients with focal parenchymal hemorrhages following TBI and SAH (93–97). These physiology studies contribute to better refine future treatments of brain edema, according to the specific pathology.

### Inflammation and Oxidative Stress

Using CMD has allowed the exploration of cytokine and chemokine profile after ABI (98–101), as well as to follow the dynamic changes in brain extracellular fluid of other biomarkers of inflammation (102), oxidative stress (NAA, isoprostane) (103, 104), and endothelial dysfunction (nitric oxide) (105), which may also be potential surrogate endpoints for interventional studies (58). Two recent scoping systematic reviews have addressed the potential value of microdialysis cytokines in severe TBI and poor-grade SAH (106, 107): although preliminary studies support feasibility of measurements and associations of CMD cytokines with tissue and neurophysiologic outcomes, evidence is very limited and further larger studies need to be conducted.

### Neurodegeneration

Markers of axonal degeneration—such as tau, β-amyloid, neurofilament light-chain (NfL), and neurofilament heavy chain (NfH)—have been the focus of recent clinical investigation, often in combination with magnetic resonance imaging, to better characterize posttraumatic axonal injury acutely in the intensive care unit (108–113). Preliminary data also established a potential link between tau protein and early brain injury following SAH (114, 115). Providing the reproducibility of these biomarkers is confirmed in larger scale studies, such approach holds great promise for early prognostication (to complement clinical and radiological information) and for a pathology-based patient selection to optimize future pharmacological interventional studies.

Table 2 summarizes main results of clinical CMD studies and their potential implications and clinical utility.

**Table 2:** Summary of clinical CMD studies.

Studies	Summary of main results	Clinical utility	Reference
**Observational studies**			

Glycemic control	Tight (4–6 mM) vs. moderate (6.1–8 mM) glycemic control is associated with more episodes of low glucose_CMD_	Management of insulin	(11, 12, 26–31)
Cerebral perfusion	Cerebral hypoperfusion is associated with increased cerebral metabolic distress (high L/P_CMD_/low gluc_CMD_)	Early ischemia detection	(37, 42, 43)
		Targeted CPP therapy	
Hemoglobin level	Anemia (Hgb <9 g/dL) is associated with increased cerebral metabolic distress	Management of RBCT	(46, 47, 50, 51)
Oxygen therapy	NBHO (2–4 h) is associated with improved LPR_CMD_	Targeted management of PaO_2_/FiO_2_	(57–59)
	NBHO benefit mostly when baseline lactate_CMD_ >3.5 mM		
	HBOT is associated with improved L/P_CMD_		

**Interventional studies**			

NOS inhibitors	NOS inhibition (i.v.) does not affect cerebral metabolism	Potential for CMD biomarkers to be used as surrogate efficacy endpoints in phase II clinical trials	(61)
rh IL-1 ra	rh IL-1ra (i.v.) does not affect cerebral metabolism		(62)
Hypertonic lactate	Hypertonic lactate (i.v.) is associated with glucose_CMD_ increase		(66, 67)
Succinate	Succinate (i.c.) is associated with reduced cerebral metabolic distress		(65)

**Mechanistic studies**			

Seizures	Electrographic seizures are associated with increased cerebral metabolic distress	Monitoring and testing the efficacy of future interventions targeted at reducing seizure and CSD	(84)
CSD	CSD are associated with low glucose_CMD_		(86, 87)
Brain edema	Cellular edema is associated with increased ${\text{Na}}_{\text{CMD}}^{+}$, ${\text{K}}_{\text{CMD}}^{+}$, and taurine_CMD_	Targeted therapy of brain edema based on disease pathology	(90–92, 96, 97)
	Vasogenic edema is associated with increased MMP_CMD_		
Neuroinflammation	Identification of several cytokines (including IL-1ra, IL-6, IL-8, and TNF-α) involved in the complex inflammatory cascade following acute brain injury	Development of therapeutics targeted at attenuating the inflammatory cascade	(106, 107)
Neurodegeneration	Relationship of tau and NfL with MRI axonal degeneration and patient outcome	Characterization of disease neuropathology	(108, 109)
		Patient selection for interventional studies targeted at reducing neurodegeneration	

*CMD, cerebral microdialysis; CPP, cerebral perfusion pressure; CSD, cortical spreading depression; FiO_2_, fraction of inspired oxygen; HBOT, hyperbaric oxygen therapy; Hgb, hemoglobin; i.c., intracerebral; IL, interleukin; i.v., intravascular; L/P lactate/pyruvate ratio; MMP, matrix metalloproteases; MRI, magnetic resonance imaging; NBHO, normobaric hyperoxia; NfL, neurofilament light chain; NOS, nitric oxide synthase; PaO_2_, arterial partial pressure of oxygen; ra, receptor antagonist; RBCT, red blood cell transfusion; rh, recombinant human; TNF, tumor necrosis factor*.

## Implementation in the Intensive Care Unit

Barriers to the widespread implementation of CMD are numerous, including costs, human resources, and the complexity of the technique (especially with respect to 100 kDa catheters) (1). These barriers may explain why CMD monitoring is still not in use in the majority of centers, as judged by a recent National survey on multimodal monitoring conducted in the UK (116). Recent consensus guidelines for the use of CMD in acute brain pathologies (2, 15) and the increased application of CMD in other acute contexts, e.g., anoxic-ischemic (117) or hepatic encephalopathy (118), may contribute to a broader implementation of this technique. The future of CMD is constantly evolving: technical refinements and the potential for automated near real-time continuous measurements may increase the performance and the accuracy of the technique (119–121), thereby facilitating the utilization in the intensive care unit.

## Conclusion

Cerebral microdialysis is an important neuromonitoring tool that is increasing used at the bedside in combination with ICP and PbtO2 to guide therapy individually in brain-injured patients. Recent consensus on microdialysis monitoring may help optimizing protocols for microdialysis implementation in neurocritical care. Over the last decade, clinical investigation using microdialysis have contributed to better understand pathogenic mechanisms involved in secondary brain damage, such as cerebral edema, energy dysfunction, cortical spreading depression, neuroinflammation, and help refining novel therapeutic approaches, and drug effects on downstream targets. Future improvements of CMD technology may further enhance applicability.

## Author Contributions

LC drafted the manuscript and Table 1. PB drafted the manuscript and the figures. MO drafted and revised the manuscript, and drafted the Figures and Table 2.

## Conflict of Interest Statement

The authors declare that the research was conducted in the absence of any commercial or financial relationships that could be construed as a potential conflict of interest.
